# Regulation of pro and anti-inflammatory signaling molecules in effect of Withania Somnifera root extract treated sleep deprivation induced Wistar rats

**DOI:** 10.6026/97320630016856

**Published:** 2020-11-30

**Authors:** K Suganya, E Kayalvizhi, K Senthil Kumar, R Muthulakshmi, R Yuvaraj

**Affiliations:** 1 Sri Ramachandra Medical College, Porur, Chennai, Tamil Nadu, India; 2 Meenakhshi Academy of Higher Education and Research, Enathur, Kancheepuram, Tamil Nadu, India; 3 Madha Medical College, Kundrathur, Chennai, Tamilnadu, India; 4 Bharath University, Selaiyur, Chennai, Tamilnadu, India

**Keywords:** Sleep Deprivation (SD), Withania Somnifera (WS), modified multiple platform method, pro-inflammatory markers, anti-inflammatory marker

## Abstract

Sleep plays an imperative role in maintaining good health. Sleep along with circadian cycle wields strong regulatory control over immunity. Sleep deprivation (SD) is a threat to health developing several immunological disorders. The medicinal plant Withania somnifera (WS) root extract is widely used for its immuno-modulatory properties. Therefore, it is of interest to assess the effect of WS root extract on pro and anti-inflammatory signalling in SD rats. 24 male Wistar rats (120-150g) were divided into 4 groups with 6 animals in each. The groups were divided such that Group I - cage control, Group II - large platform control, Group III - sleep deprived & Group IV - WS treated SD rats. RT-PCR based mRNA expression analysis of pro inflammatory (IL-1β, IL-6, MCP-1, TNF-α) and anti-inflammatory marker (IL-10) in the cortex of control and SD rats were completed. Concurrent protein expression analysis was completed using western blot. Data was analyzed using one-way ANOVA and Duncan's multiple range test in SPSS software version 20. Data showed elevation of pro-inflammatory markers and depression of IL-10. Thus, WS down regulated the pro-inflammatory and up-regulated the anti-inflammatory molecules, which can be further considered towards the treatment of sleep deprivation induced inflammatory diseases.

## Background

Sleep plays an imperative role in maintaining good health. World sleep society too affirmed that "When Sleep is Sound, Health and Happiness is Abound [1]. Sleep is a chief controller of immunological homeostasis and is vital to recover from illness. An increasing body of literature is also insisting its importance for holding a good defense system [2]. The interaction between the sleep and immune system is mediated by shared signals such as neurotransmitters, hormones and cytokines. Lack of sleep may diminish the immune response by altering those signaling molecules [2]. Sleep deprivation is a symbol of stress, which can have risky physiological consequences affecting all body systems including brain [3]. Various immunological disorders arise from sleep loss, which in turn increases the receptiveness for infection. In addition to humans sleep deprivation in animals also leads to systemic infection, afebrile septicemia and invasion of microorganisms etc. [4] Withania somnifera, a member of Solonaceae family is extensively used for its antioxidant, immunomodulatory, anti-inflammatory and anti-tumor properties. It is also considered to be an adaptogen, facilitating the ability to withstand stress [5 - check with author]. Nowadays, the application of immunomodulators is the practice of modern method for the correction of immunodeficiency. Most of the immunomodulators corrects the immune system by regulating the cytokine production by activated target cells [6-8]. According to Ganju et al. (2003) [9], immunomodulation using medicinal plants can provide an alternative to conventional therapy for a variety of diseases particularly in immunological disorders. Therefore, it is of interest to assess the effect of WS root extract on pro and antiinflammatory signaling in induced sleep deprivation rat models.

## Materials & Methods:

The study was done in the Department of Physiology, Meenakshi Medical College & Hospital, Enathur, Kanchipuram. Proper Ethical clearance was obtained from the CPCSEA (IAEC No: 007/2017). 24 male Wistar rats weighing 120-150g were used for the study. They were divided into 4 groups with 6 animals in each group. (Group I - cage control, Group II - large platform control, Group III - sleep deprived group & Group IV - WS treated SD group). Animals were deprived sleep for one week using a modified multiple platform method. Pro and anti-inflammatory signalling molecules and growth factors were measured among the experimental groups. Gene expression analysis was done using Real Time PCR and protein expression analysis was done using western blot analysis. Statistical analysis was done by one-way ANOVA and Duncan's multiple range tests.

## Sleep Deprivation Technique:

### Modified Multiple Platform Method:

The rats were deprived of sleep for one week using modified multiple platform method (7cms) [10 - check with author]. They were placed on the top of a small platform which is mounted on a customized water tank filled with water of approximately 1cm. The water tank has 12 circular platforms fixed inside which is designed for free movement of rats. When the rat goes into sleep it loses its muscle tone and fall off from the platform into the water, which then climbs up back and awake from sleep. Large Platform Control group rats (Group II) were left free on a large platform (14 cms) in the same environment where sleep deprivation was performed.

### Ethanolic extract of Withania somnifera:

The roots of WS were air dried and powdered and the ethanolic extract was prepared with 95% ethanol for 12 hours in Soxhlet extractor. The extract obtained was concentrated using rotary vaccum evaporator at 40° to 60°C. The semisolid extract, which is concentrated, was stored in refrigerator at 2-8°C. For experimentation, the extract was dissolved in DMSO (Dimethyl sulphoxide) and administered orally to animals for 30 days with a dosage of 400mg/kg bw [11 - check with author, 12].

### Estimation of pro and anti-inflammatory signaling molecules and growth factors:

The mRNA expression of pro inflammatory markers (IL-1β, IL-6, MCP-1, and TNF-α), anti-inflammatory marker (IL-10) in the cortex of control and sleep deprived rats was done using real time PCR [13]. Protein expression was done using western blot analysis [14 - check with author].

## Statistical analysis:

The data obtained were subjected to statistical analysis using oneway analysis of variance (ANOVA) and Duncan's multiple range test to assess the significance of individual variations between the control and treatment groups using a computer based software (Graph Pad Prism version 5). In Duncan's test, the significance was considered at the level of p<0.05.

## Result:

### Effect of W.somnifera on mRNA expression of IL-1β, IL-6, MCP-1, and TNF-α in the cortex of control and sleep deprived rats:

In the Figures 1, 2, 3 & 4(see PDF) the Group III (SD rats) showed a significant up regulation of pro-inflammatory markers IL-1β, IL-6, MCP-1, and TNF-α mRNA expression in the brain cortex when compared to Group I (Cage Control rats) and Group II (Large Platform Control rats). Similarly in Group II (Large Platform Control rats) the pro-inflammatory markers showed a significant up regulation when compared to Group I. Whereas the Group IV, which is treated with W.somnifera root extract significantly, showed a down-regulated pro inflammatory cytokines when compared with Group II and Group III but the significance was not observed with Group I.

### Effect of W.somnifera on mRNA expression of the antiinflammatory marker IL-10 in cortex of control and sleep deprived rats:

In the Figure 5(see PDF) the Group III (SD rats) showed a significant down regulation of mRNA expression of the anti-inflammatory marker, IL-10 in the brain cortex when compared to Group I (Cage Control rats) and Group II (Large Platform Control rats). Similarly in Group II (Large Platform Control rats) IL-10 showed a significant down regulation when compared to Group I. Whereas the Group IV, which is treated with W.somnifera root extract significantly, showed an up regulation of IL-10 when compared with Group II and Group III but the significance was not observed with Group I.

### Effect of W.somnifera on the protein expression of the proinflammatory markers (IL-1 β, IL-6, MCP-1 and TNF-α) in the cortex of control and sleep deprived rats:

The protein expression of the pro-inflammatory markers IL-1β, IL-6, MCP-1, and TNF-α mRNA shown in the Figures 6, 7, 8 & 9(see PDF) in the brain cortex showed a significant up regulation in group III (SD rats) when compared to Group I (Cage Control rats) and Group II (Large Platform Control rats). Similarly protein expression in Group II (Large Platform Control rats) showed a significant up regulation when compared to Group I. Whereas the Group IV which is treated with W.somnifera root extract significantly showed a down regulated pro inflammatory cytokines when compared with Group II and Group III but the significance was not observed with Group I.

### Effect of W.somnifera on the protein expression of the antiinflammatory marker (IL-10) in the cortex of control and sleep deprived rats:

The protein expression of the anti-inflammatory marker shown in the Figure 10(see PDF) in the brain cortex showed a significant down regulation in-group III (SD rats) when compared to Group I (Cage Control rats) and Group II (Large Platform Control rats). Similarly protein expression in Group II (Large Platform Control rats) showed a significant down regulation when compared to Group I. Whereas the Group IV, which is treated with W.somnifera root extract significantly, showed an up regulation of IL-10 when compared with Group II and Group III but the significance was not observed with Group I.

## Discussion:

Sleep is hypothesized to be a restorative process that is important for the appropriate functioning of the immune system. Sleep disorders are associated with decline in innate and cellular immunity and also with alterations in the complex cytokine network. Cytokines are regulators of host responses to infection, immune responses, inflammation and trauma. Some cytokines act to make disease worse (pro-inflammatory), whereas others serve to reduce inflammation and promotes healing (anti-inflammatory). In our study we explored the possibility that sleep deprivation may hassle major changes in the immune system by inducing inflammation. Rats were subjected to one week of sleep deprivation and the levels of pro-inflammatory cytokines (IL-1beta, IL-6, MCP-1 and TNF-alpha), an anti-inflammatory cytokine (IL-10) were measured among the experimental groups. In our findings, the levels of the pro-inflammatory cytokines were significantly elevated and anti-inflammatory cytokine IL-10 was significantly reduced in sleep-deprived rats when compared to control group.

Our results point out that sleep deprivation induced an inflammatory response. The possible mechanism might be due to the action of stress induced effect of glucocorticoids on antigen presenting cells (APCs) and T helper cells in up-regulating their production of pro-inflammatory cytokines and down-regulating their production of anti-inflammatory cytokines when the rats were exposed to sleep deprivation [15]. Activation of toll-like receptors (TLRs) present on monocytes, macrophages and dentritic cells results in the gene transcription that stimulates nuclear factor kappa - beta (NF-Kβ) which in turn leads to the production of inflammatory cytokines. In a recent study done on multi factorial high stress military ranger training which included sleep deprivation, a significantly increased monocyte TLR expression was observed throughout the training course with transiently increased TNF-α level. Thus it is evident in our study that sleeps deprivation led to an alteration in the innate functional cellular immunity by increasing the gene expression of pro-inflammatory cytokines. Also the increase in the pro-inflammatory cytokines could be due to the increased free radical production with decreased anti-oxidant enzymes following sleep deprivation, which was evident from our previous study [16]. Chennaoui et al. 2014 [17] showed that the IGF system depresses the pro-inflammatory cytokine signaling by increasing the antiinflammatory cytokine IL-10 expression. The anti-inflammatory effect of IGF is through the down regulation of TLR-4 signaling and subsequent reduction of NF-KB. Growth hormone is the potent stimulator of IGF secretion and several researchers have predicted that 75% of growth hormone secretion occurs during sleep. In our study, the elevation of pro-inflammatory cytokines might be due to the decline in the basal secretion of growth hormone following sleep deprivation, which is mediated through IGF secretion. The up-regulation of pro-inflammatory cytokines in our study following sleep deprivation is parallel to the findings of the study done by Nakajima et al. 2010 [18] who concluded that sleep deprivation might affect the immune homeostasis by altering its mediators.

In the group treated with Withania somnifera, the levels of pro inflammatory markers were significantly reduced and it also up regulated the anti inflammatory marker IL-10. The active components such as Withaferin A and Withanolide E [5 - check with author] showed a significant modulation of immune reactivity in our study. These data shed a new light on the reversal effect of Withania Somnifera in sleep deprivation induced immunomodulatory changes.

## Conclusion

We show that WS root extract down regulated the proinflammatory and up-regulated the anti-inflammatory molecules in sleep deprivation induced Wistar rats for further consideration in the treatment of inflammation-linked diseases.
